# Effects of Air Pollutants on Airway Diseases

**DOI:** 10.3390/ijerph18189905

**Published:** 2021-09-20

**Authors:** Yun-Gi Lee, Pureun-Haneul Lee, Seon-Muk Choi, Min-Hyeok An, An-Soo Jang

**Affiliations:** Department of Internal Medicine, Soonchunhyang University Bucheon Hospital, 170 Jomaru-ro, Wonmi-gu, Bucheon 14584, Gyeonggi-do, Korea; dldbs0716@naver.com (Y.-G.L.); gksmf5637@naver.com (P.-H.L.); shcy0426@naver.com (S.-M.C.); qsef7474@naver.com (M.-H.A.)

**Keywords:** air pollutants, airway disease, asthma, COPD

## Abstract

Air pollutants include toxic particles and gases emitted in large quantities from many different combustible materials. They also include particulate matter (PM) and ozone, and biological contaminants, such as viruses and bacteria, which can penetrate the human airway and reach the bloodstream, triggering airway inflammation, dysfunction, and fibrosis. Pollutants that accumulate in the lungs exacerbate symptoms of respiratory diseases such as asthma and chronic obstructive pulmonary disease (COPD). Asthma, a heterogeneous disease with complex pathological mechanisms, is characterized by particular symptoms such as shortness of breath, a tight chest, coughing, and wheezing. Patients with COPD often experience exacerbations and worsening of symptoms, which may result in hospitalization and disease progression. PM varies in terms of composition, and can include solid and liquid particles of various sizes. PM concentrations are higher in urban areas. Ozone is one of the most toxic photochemical air pollutants. In general, air pollution decreases quality of life and life expectancy. It exacerbates acute and chronic respiratory symptoms in patients with chronic airway diseases, and increases the morbidity and risk of hospitalization associated with respiratory diseases. However, the mechanisms underlying these effects remain unclear. Therefore, we reviewed the impact of air pollutants on airway diseases such as asthma and COPD, focusing on their underlying mechanisms.

## 1. Introduction

Air pollutants include toxic particles and gases emitted in large quantities from many different sources, including vehicles and factories [1]. Indoor pollutants include smoke from tobacco, cooking, and the burning of wood and other materials in stoves and fireplaces, as well as dust particles disturbed during cleaning and outdoor particles that infiltrate the indoor environment [1]. Major pollutants include particulate matter (PM), ozone, nitrogen dioxide (NO_2_), and sulfur dioxide (SO_2_). Biological contaminants such as viruses, bacteria, animal dander and cat saliva, house dust mites (HDMs), cockroaches, and pollen can exacerbate allergic reactions and airway diseases, such as asthma, allergic rhinitis (AR), and hypersensitivity pneumonitis [2,3,4].

Air pollution negatively impacts human health and increases the burden of disease and demand for healthcare services. Air pollution is the fourth largest contributor to disability-adjusted life years (DALYs) and mortality, and was responsible for approximately 200 million DALYs and 6.67 million deaths in 2019 according to a Global Burden of Disease analysis [5]. Air pollution was responsible for an estimated 5 million deaths worldwide in 2017, 70% of which were due to outdoor air pollution [5]. Children living in polluted areas are more likely to have airway hyperresponsiveness (AHR) than those living in less polluted areas [6]. Exposure to ambient PM ≤ 2.5 μm in aerodynamic diameter (PM2.5) is the most important environmental factor in the global burden of disease [7,8,9,10,11], with an estimated 3 million deaths worldwide being attributable to exposure to PM2.5 in 2017. The World Health Organization (WHO) estimates that 92% of the world’s population live in areas where the annual mean PM2.5 exceeds 10 μg/m^3^, which is the WHO cut-off value for poor air quality [12].

PM is the principal component of many indoor and outdoor air pollutants, and can include solid and liquid particles of various sizes. PM may be emitted from cars, motorbikes, buses, and trucks, as well as from heating furnaces, power plants, and factories [13]. PM can be coarse, fine, or ultrafine, and is often a complex mixture of materials with a carbonaceous core and other constituents, such as organic compounds, acids, and fine metal particles [14]. When inhaled, particles larger than 10 μm in size generally become trapped in the nose or throat and do not enter the lungs [15,16]. Particles smaller than 10 μm but larger than 2 μm may enter the tracheobronchial system, but are removed via mucociliary clearance. Smaller particles can penetrate the airway and reach the alveolar region of the lung, thus exacerbating airway and respiratory diseases such as asthma and chronic obstructive pulmonary disease (COPD). In the alveolar region, cytokines and chemokines direct neutrophils and macrophages to foreign particles, which are engulfed by phagocytosis [15,16].

Ambient ozone is another environmental air pollutant that has a substantial impact on human health [17,18]. Ozone is highly reactive and oxidizes proteins and lipids in the fluid-lined compartment of the lung [17,18]. Epidemiological data suggest that individuals with chronic airway diseases, such as asthma or COPD, are particularly sensitive to ozone exposure and may exhibit increased morbidity and a higher risk of mortality in response to ozone [17,18]. Ozone is one of the most toxic photochemical air pollutants, and may increase mortality and hospital admission rates by exacerbating respiratory and cardiovascular diseases [19]. Decreased quality of life and life expectancy are often linked to air pollution. Recently, a causal relationship between asthma and proximity to road-traffic-related pollution was reported in 15% of all asthma episodes [20]. Importantly, acute effects of air pollution increase the likelihood of hospitalization [21]. The chronic effects of air pollution have been quantified by estimating the number of living years lost due to long-term exposure to air pollution [21].

The adverse effects of PM on health may also be exacerbated by ozone. Ozone may induce damage to the lung epithelium and interfere with the inflammatory response [22,23,24,25,26], as well as mucociliary clearance [26]. Decreasing the levels of PM ≤ 10 μm in the aerodynamic diameter (PM10), PM2.5, and ozone are important for improving human health and avoiding hospital visits and admissions, as well as for minimizing the economic costs associated with healthcare.

Ambient air pollution is one of the greatest environmental threats to human health worldwide, and it has both acute and long-term effects [27]. Pollutants can also have adverse effects on health via oxidant-mediated cellular damage [28], reactive oxygen species (ROS), oxidative stress, and innate and adaptive immune responses.

PM size may be particularly important for predicting the effect of pollutants on airway inflammation. PM size may be negatively correlated with the level of toxicity in the lungs. Therefore, PM2.5 may be more toxic than PM10 [29]. As oxidative stress and chronic airway inflammation are major factors in chronic airflow limitation in patients with COPD, air pollution may exacerbate COPD [30].

Despite the increasing experimental data on air pollutant toxicity, more studies are needed to understand the relationship between air pollutants and airway diseases. A meta-analysis demonstrated a significant correlation between exposure to various air pollutants and the incidence of childhood asthma [31,32,33], and also suggested that there may be a causal relationship between air pollution (i.e., PM2.5 and NO_2_) and COPD development. Here, we review the impact of air pollutants on airway diseases such as asthma and COPD, focusing on the underlying mechanisms.

Bioaerosol is a particulate mixture of solid and semi-solid matter combined with biotic matter such as pollens, microbes and their fragments [34]. They are transmitted through the air with a particle size ranging from 0.001 nm to 100 μm. The pathophysiological effects of these bioaerosol pollutants depend on their size, concentration, physiochemical properties and size distribution [34]. Due to their micro- to nano-scale size, bioaerosol scan easily deposit in various parts of the body via the lungs and circulatory system. This deposition can cause a number of health complications involving from single organ to an entire organ system [35]. Bioaerosol exposure is associated with adverse health effects, including allergies, acute toxic effects, infectious diseases, and cancer [36,37]. Human exposure to bioaerosols has been associated with a range of acute and chronic adverse health effects and diseases. The most commonly reported are respiratory diseases such as rhinitis, asthma, bronchitis and sinusitis, through both atopic and non-atopic allergic mechanisms as well as non-allergic pathways [36].

### 1.1. The Effects of Air Pollutants on Asthma and COPD, Upper Airway Disease

Respiratory diseases that involve the airway (e.g., the nose, pharynx, trachea, bronchi, and alveoli) are very common and have various symptoms, including cough, chest pain, shortness of breath, and respiratory failure; moreover, they can be fatal [38]. Asthma is a common, chronic respiratory disease affecting 8.0% of adults and 7.0% of children. The prevalence of post-bronchodilator COPD is 12.16% (10.91–13.40%). The pooled prevalence of COPD was 15.70% (13.80–18.59%) in men and 9.93% (8.73–11.13%) in women [39].

Air pollution is one of the most important environmental factors affecting public health, due to its effects on the respiratory system [40]. Air pollution can interfere with defense mechanisms in the lung, weaken the body’s immune response [41], and trigger oxidative stress and inflammation [42,43]. Air pollution is defined as the presence of aerial substances that are harmful to humans, and is associated with a higher risk of premature death due to cardiovascular diseases (e.g., ischemic heart disease and stroke), asthma, COPD, lower respiratory tract infections, and lung cancer [31,40]. Ozone has a strong smell and irritates the respiratory system, resulting in swelling of the throat, discomfort in the chest, coughing, sputum production, and even emphysema with long-term exposure [44]. Nitric oxide may generate photochemical smog and has acute toxic effects on the human lungs. The short- and long-term effects of PM10, PM2.5, and SO_2_ on lung function, disease morbidity, and mortality have been described in detail [23,40,45]. Air pollution is associated with various respiratory and non-respiratory diseases including asthma, COPD, pneumonia, lung malignancies, heart disease, stroke, dementia, and diabetes [46,47,48,49,50].

Asthma is a chronic respiratory disease characterized by varying degrees of airflow obstruction, AHR, and airway inflammation. The current data suggest that air pollution has a negative impact on asthma outcomes in both adults and children [51,52,53,54]. Outdoor pollutants may induce asthma symptoms and impair lung function. Smoking tobacco is associated with poor control of asthma symptoms, whereas passive smoking increases the risk of asthma exacerbations and respiratory symptoms, as well as healthcare needs [51,52,53,54]. Indoor pollutants from other sources, such as heaters and molds, may also negatively impact the course of asthma. Coordinated global efforts will be necessary to reduce exposure to air pollutants, and improve outcomes for adults and children with asthma [51,52,53,54]. Many studies have shown that outdoor air pollution is correlated with asthma hospitalizations [51,52,53,54]. Increased air pollution also correlates with an increase in asthma cases and promotes exacerbation [55,56,57,58,59].

The prevalence of asthma in different countries varies from ~1 to 18% of the population [60,61,62,63]. The current evidence suggests that 13% of pediatric asthma cases worldwide may be attributable to traffic-related air pollutants (TRAPs), and that air pollution has a negative impact on asthma outcomes in both adult and pediatric patients [64]. Increasing evidence indicates that both outdoor and indoor air pollution contribute to the development of asthma. Numerous cross-sectional studies have suggested that poor air quality plays a role in asthma [60,61,62,63]. A recent meta-analysis demonstrated a link between the development of asthma and increased exposure to TRAPs, particularly PM2.5, PM10, NO_2_, and black carbon [33].

**Figure 1:**
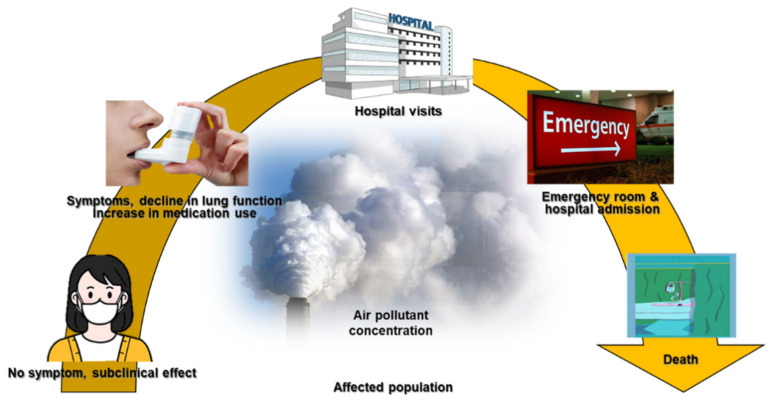
The impact of air pollutants on respiratory diseases.

The long-term effects of air pollution on asthma were summarized in an American Thoracic Society workshop report, which indicated that long-term exposure to air pollution was a cause of childhood asthma. However, there was insufficient evidence to draw a similar conclusion regarding adult asthma [65]. Many studies have described correlations between short-term exposure to outdoor air pollutants and various aspects of asthma, including symptom control [66], lung function [67], medication dose [68,69], outpatient visits [70,71], asthma exacerbations [72,73], emergency room visits [74], hospitalizations [75,76], length of hospital stay [77], and mortality rate [78] (Figure 1).

COPD is a disease characterized by exhaustion that frequently affects smokers and involves progressive respiratory symptoms, such as dyspnea and cough with sputum. These symptoms hinder daily activities and exercise, and reduce patients’ quality of life [79]. COPD incurs significant healthcare costs, and increases morbidity and the risk of mortality [80]. Acute exacerbations in patients with COPD lead to worsening symptoms requiring additional therapy and hospitalization [81].

The most common cause of COPD is smoking; some cases are also due to air pollution and genetics [82]. Poorly ventilated cooking fires, often using coal or biomass fuels such as wood, lead to indoor air pollution and are a common cause of COPD in developing countries [83]. Such fires are used by nearly 3 billion people for cooking and heating, and adverse effects on health are more frequent in women due to their higher levels of exposure [84,85]. These fires are the main source of energy in 80% of all homes in India, China, and sub-Saharan Africa [85].

People who live in large cities are more likely to develop COPD than those who live in rural areas [86]. However, although urban air pollution contributes to exacerbations, its role in COPD is unclear [84]. Areas with poor outdoor air quality, for example, due to the high levels of exhaust fumes, generally show a higher prevalence of COPD [85]. However, the overall effect of poor air quality on disease relative to that of smoking is considered to be small [86]. The results of a meta-analysis showed that a 10 μg/m^3^ increase in PM2.5 was associated with an increased incidence of COPD (pooled hazard ratio (HR), 1.18; 95% confidence interval (CI), 1.13–1.23) [31]. A 10 μg/m^3^ increase in NO_2_ was associated with a marginal increase in the incidence of COPD (pooled HR, 1.07; 95% CI, 1.00–1.16) [31]. In contrast, the level of PM10 had no significant impact on the incidence of COPD (pooled HR, 0.95; 95% CI, 0.83–1.08), although this conclusion was based on few studies [31]. Long-term exposure to PM2.5 and NO_2_ may be associated with an increased incidence of COPD [31]. Epidemiological data indicate that individuals with chronic inflammatory diseases, such as asthma or COPD, are hypersensitive to ozone and thus have a higher risk of morbidity and mortality [18].

Several epidemiological studies have shown that air pollutants exacerbate airway diseases such as AR, asthma, bronchitis, and COPD. Pollutants such as TRAPs also have negative effects on other upper airway diseases such as AR and non-AR, sinusitis, and otitis media [87]. Increasing evidence suggests that PM, photochemical pollutants, and ozone are also linked to the development of upper airway diseases [87]. Young children and individuals who are obese are particularly susceptible to these conditions [87]. ROS, apoptosis, and inflammation are all involved in the pathophysiological etiology of upper airway diseases [87]. Although the data conflict, and controlled prospective studies are needed to determine the relevant mechanisms and risk factors, traffic fumes and tobacco smoke are major factors exacerbating upper airway diseases [87].

Protease-activated receptor-2 (PAR-2)-modulated TJs may also be involved in the pathogenesis of chronic airway diseases. PAR-2 may downregulate the expression of ZO-1 and CLDN1, which are involved in epithelial barrier dysfunction in patients with AR [88]. AR is associated with increased epithelial permeability. In addition, histamine and type-2 inflammation are responsible for TJ dysfunction. Epithelial barrier dysfunction promotes the transepithelial movement of allergens, increased sensitivity to allergens, and allergen-induced mast cell degranulation, even in a noninflammatory environment [89].

Interestingly, the levels of mucin 1 (MUC1) in the nasal epithelia of patients and rats with AR are significantly reduced. MUC1 deficiency exacerbates AR symptoms and nasal epithelial characteristics typical of AR. Depletion of MUC1 suppresses the expression of epithelial cell connection protein, suggesting that MUC1 deficiency is linked to AR pathogenesis. Therefore, MUC1 may be a promising therapeutic target for AR [90]. Patients with AR also exhibit altered epithelial barrier function, including alterations in mucus production, antimicrobial defense, the microbiome, and the immune response [91,92].

### 1.2. Airway Toxicity Mechanisms Related to Air Pollutants

**Figure 2:**
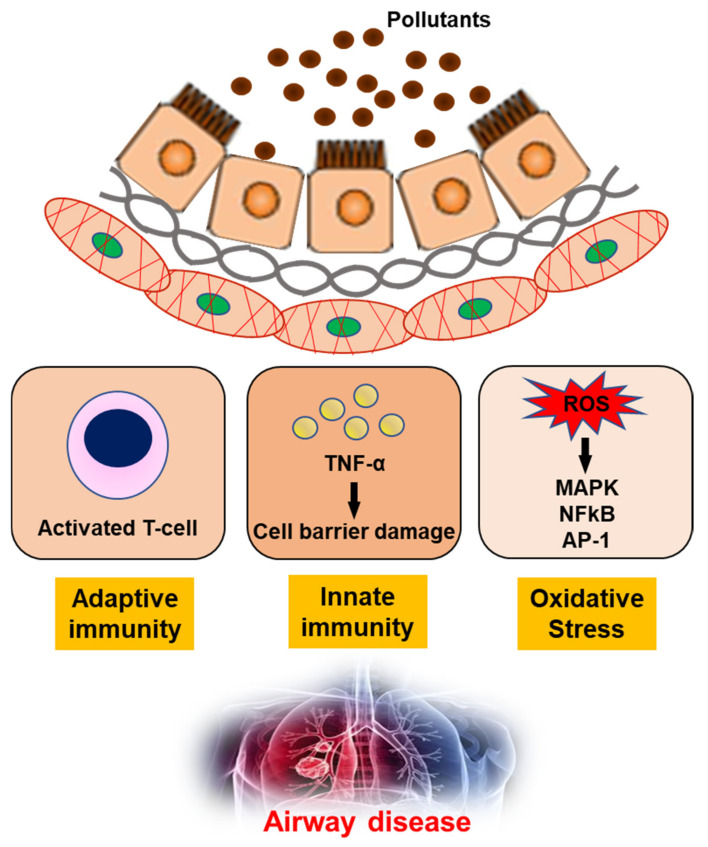
The mechanism of air pollutants for airway disease. TNF-α; Tumor necrosis factor-α, ROS; reactive oxygen species, MAPK; mitogen-activated protein kinase, NFkB; nuclear factor kappa-light-chain-enhancer of activated B cells, AP-1; Activator protein 1.

The impact of air pollution, especially on population health, cannot be underestimated and urgently needs to be addressed [93]. In-depth studies of the underlying mechanisms, together with clinical, imaging and molecular biology data, have shown that exposure to air pollution promotes the development of airway diseases [94,95,96,97]. Pollutants can generate oxidant-mediated cellular damage [28] via ROS production, other types of oxidative stress, and innate and adaptive immune responses that may have adverse effects on health (Figure 2).

Ozone is highly reactive, and oxidizes proteins and lipids in the fluid-lined compartment of the lung. This initiates inflammation and increases lung permeability, via cytotoxic mediators including pro-inflammatory cytokines, ROS, and nitrogen intermediates such as peroxynitrite [17]. The primary targets for ozone are the distal structures of the lung, including the terminal bronchioles, bronchiole–alveolar duct junction, and proximal alveolar regions [17]. Acute inhalation of ozone causes structural alterations in the lung, including disruption of the alveolar epithelial barrier, which lead to alveolar epithelial type II cell hypertrophy and hyperplasia [93,98]. The recruitment of inflammatory cells into the lung following ozone exposure can also damage tissue via the release of toxic mediators (e.g., cytokines, ROS, nitrogen species, and proteolytic enzymes) from activated macrophages and neutrophils [93,98].

Ozone is a reactive oxidant, and pycnogenol is a mixture of flavonoid compounds from pine tree bark that have antioxidant properties. When ingested, pycnogenol may increase the levels of antioxidant enzymes and decrease those of nitrogen species, suggesting that antioxidants minimize the effects of acute ozone exposure [99]. Antioxidant responses may protect BALB/c mice exposed to ozone from a range of oxidants [98]. Proliferating cell nuclear antigen (PCNA) is a component of one of the multiprotein complexes expressed during cell proliferation. Ozone can induce alveolar epithelial cell proliferation in a dose-dependent manner, and alveolar epithelial cell proliferation is correlated with airway obstruction [93]. Ozone-induced epithelial injury in the alveoli results in a dose-dependent increase in alveolar epithelial cell proliferation and a dose-dependent decrease in PCNA levels in nasal skin. This finding suggests that cell proliferation responses in nasal skin and the airway differ after short-term ozone exposure, which may reflect differences in epithelial cell damage and repair processes [100]. Short-term exposure to high concentrations of ozone can increase AHR. This increase in AHR does not persist during chronic ozone exposure, indicating that airway remodeling and adaptation following repeated exposure to air pollutants can provide protection against AHR [101].

Allergic airway diseases are linked to exposure to atmospheric pollutants. This may be a factor in the increasing prevalence of asthma. Little is known about the combined effect of ozone and diesel exhaust particles (DEPs) on the development and exacerbation of asthma. Co-exposure to ozone and DEPs has an additive effect on AHR, exerted via the modulation of interleukin (IL)-4 and interferon (IFN)-γ, suggesting that DEPs amplify the Th2 immune response [102]. The AHR induced by acute inhalation of ozone depends on the concentration of ozone and duration of exposure. Airway obstruction is induced following ozone exposure in a concentration-dependent manner and persists for at least 72 h [103,104].

Approximately 5% of all individuals with asthma have refractory asthma (RA), i.e., have difficulty controlling the disease. Patients with RA (*n* = 82) within the metropolitan area of Seoul and Gyeonggi Province constituted 3.7% of all the individuals with asthma enrolled in our cohort between 2005 and 2009 (*n* = 2298). In winter, a 1 °C decrease in ambient temperature and 1 ppb increase in SO_2_ concentration on Lag day 1 were associated with 14.8% (95% CI: 0.9–26.7) and 19.7% (95% CI: 3.3–38.7) increases in the risk of RA exacerbation among nonsmokers, respectively. Similar associations were observed for Lag day 2. During the winter, exposure to low temperature and increased SO_2_ concentrations are positively correlated with the occurrence of acute RA exacerbations 1–2 days later [105].

PM is the main component of most air pollutants. PM includes a range of particle sizes, such as coarse, fine, and ultrafine particles. Individuals are primarily exposed to PM via inhalation. The inhalation of PM exacerbates respiratory symptoms in patients with chronic airway diseases, but the mechanisms underlying this response remain unclear [16]. Nanoparticles (NP) may cause cell and tissue damage, leading to local and systemic inflammatory responses and adverse effects on health. The inflammasome is a major regulator of inflammation via its activation of pro-caspase-1, which cleaves pro-IL-1β into a mature form and may induce acute and chronic immune responses to NPs. Inflammasome activation was observed in the lungs of individuals with asthma following NP exposure, suggesting that targeting the inflammasome may assist in controlling NP-induced airway inflammation and hyperresponsiveness [106]. Interestingly, treatment with titanium dioxide (TiO_2_) increased the level of mRNA-encoding macrophage migration-inhibitory factor (MIF). MIF was primarily expressed in the epithelium and was elevated in lung tissue and bronchoalveolar lavage fluid from TiO_2_- compared to sham-treated rats. Carbon black and DEPs also induced the expression of MIF protein in epithelial cells [107].

DEPs can trigger AHR and inflammation. Long-term DEP exposure increased AHR, inflammation, lung fibrosis, and goblet cell hyperplasia in a mouse model [108]. The interaction between chronic inflammation and neural dysfunction in the airways suggests a link between the nervous and immune systems. Substance P, ATP, and calcitonin gene-related peptide (CGRP) levels in bronchoalveolar lavage fluid were increased in ovalbumin (OVA)-sensitized mice, and these increases were augmented in OVA-sensitized NP-exposed mice. Bradykinin, ATP, and CGRP levels were all increased in NP-exposed normal human bronchial epithelial (NHBE) cells in a dose-dependent manner. Calcium concentrations were increased in NHBE cells exposed to NPs for 8 h. These results indicate that neuroinflammation may be involved in the pathogenesis of bronchial asthma, and that NPs can exacerbate asthma via neuro-mediator release [109].

Air pollutants and obesity are important contributors to asthma. The current data indicate increased airway inflammation in DEP-exposed obese rats compared to their non-obese counterparts, indicating that DEPs and obesity may both increase asthma severity [110].

Unsurprisingly, exposure to environmental pollutants is associated with adverse respiratory outcomes. The phosphorylation of enzymes activates or deactivates many cellular processes, and is linked to the development of lung diseases such as asthma and COPD. Immunoblotting with anti-GSTP1 antibody revealed no change in GSTP1 protein levels in BEAS-2B cell lysates after treatment with TiO_2_ particles; blotting with anti-phosphoserine and anti-phosphotyrosine antibodies revealed dose-dependent decreases in phosphoserine and phosphotyrosine proteins. Exposure to foreign particles phosphorylated and dephosphorylated several proteins within epithelial cells, and the serine and tyrosine phosphorylation levels of GSTP1 decreased. These data indicate that airborne particles affect the pattern of phosphorylation of proteins involved in defense and apoptosis within the respiratory epithelium [111].

Chitinase may play a regulatory role in allergic diseases. In a mouse model, DEPs induced AHR and Ym1/2 mRNA expression via a Th2-cell-mediated response, suggesting that chitinase may play an important role in airway inflammation and responsiveness upon exposure to DEPs, and may, therefore, be involved in regulating allergic diseases [112]. PM inhalation-induced lung inflammation acts as an adjuvant to allergens or respiratory viral infection, in a process that is mediated by macrophages and epithelial cells. Alveolar macrophages play an important role in particle-induced lung inflammation by stimulating the production of IL-13 and IL-25 [113].

Tight junctions (TJs) formed of adjacent epithelial cells are an essential part of the barrier between the mucosa or skin and the external [114,115]. TJs prevent particles and pathogens from penetrating tissues, control the extracellular and paracellular flux of molecules, and help establish the apical–basolateral axis [116]. Several allergic and inflammatory diseases are reportedly associated with epithelial barrier defects and TJ disruption, including atopic dermatitis, asthma, and chronic rhinosinusitis. TJs may also play a role in asthma development [117]. However, little is known of the interplay between air pollutants and the bronchial epithelial barrier, or of the impact of PM on epithelial barrier function. PM-induced disruption of barrier function in a bronchial epithelial cell line has provided some insight into the role of epithelial barrier dysfunction in asthma [118]. When PM is inhaled, NPs such as TiO_2_ particles may cause cell and tissue damage, leading to local and systemic inflammatory responses and adverse effects on health [119]. The bronchial epithelium is constantly exposed to a wide range of environmental substances present in inhaled air, including noxious gases and anthropogenic and natural particles. These include gases and particles from car emissions, tobacco smoke, pollen, animal dander, and pathogens [120]. Therefore, the airway epithelium is an important physical barrier, as well as a modulator of allergic responses and airway inflammation [106]. Epithelial barrier dysfunction contributes to allergic inflammation and the development of asthma, because a dysfunctional barrier increases the exposure of subepithelial tissues to inhaled allergens and air pollutants. The TJ proteins known as claudins (CLDNs) are important regulators of paracellular permeability. PM exacerbates airway epithelial barrier dysfunction and leads to airway inflammation [121].

The airway epithelium is the initial barrier to external pathogens, including bacteria, viruses, chemical substances, and allergens [122,123,124]. Airway epithelial cells also have pivotal roles as coordinators of immunological defense and mediate the elimination of external pathogens from airways. When the airway epithelium is damaged, pathogens can remain in the airway and induce aberrant immunological reactions [122,123,124]. Dysregulated function of the asthmatic airway epithelium reportedly interferes with wound repair, weakens TJs, and leads to excessive proliferation and airway remodeling. This results in aberrant airway responses to external pathogens [122,123,124].

Functional studies indicate that asthmatic airway epithelia show increased sensitivity to environmental stressors and oxidative stress, thus reducing the threshold for epithelial damage [125,126,127]. Increased barrier permeability in patients with asthma increases susceptibility to allergens, reduces the threshold for epithelial damage, and activates type 2 responses [128,129,130,131]. Changes in microbial diversity within asthmatic airways have also been reported [128,129,130,131]. Furthermore, impaired epithelial barrier repair in patients with asthma can weaken inflammatory responses [132,133].

Levels of the TJ protein CLDN7 were decreased in the plasma of patients with asthma. In these patients, CLDN7 levels were indicators of lung function and the blood eosinophil concentration. CLDN7 expression was elevated in the lungs of mice with asthma, and in NHBE cells treated with HDM extracts. However, CLDN7 expression was suppressed by exposure to TiO_2_. P-AKT and p-ERK were increased in asthmatic mice and decreased in those treated with TiO_2_. Levels of p-AKT and p-ERK were decreased in NHBE cells treated with TiO_2_ and HDM extracts. Furthermore, transepithelial electrical resistance increased in NHBE cells treated with TiO_2_ or HDM extracts. However, this effect was attenuated when TiO_2_ and HDM extracts were co-administered. These data suggest that PM exacerbates airway epithelial barrier dysfunction and leads to airway inflammation [121].

The expression patterns of CLDN-4, -5, and -17 were different in the nose and lungs of mice exposed to DEPs. This suggests that similar changes occur in the cell barriers lining the upper and lower airways, raising the possibility that modulating cell barriers in the nose and lung may be useful for treating diseases of the airway [134].

N-acetylcysteine (NAC) affects the signaling pathways that are involved in apoptosis, angiogenesis, cell growth and arrest, redox-regulated gene expression, and the inflammatory response. NAC attenuated OVA-induced AHR and inflammation. Levels of CLDN18 protein in lung tissue from OVA-sensitized mice were higher than those in lung tissue from control mice, and they increased in response to treatment with NAC or dexamethasone. Treatment with NAC or dexamethasone also suppressed the OVA-induced increase in IL-1α protein levels. Although treatment with NAC increased OVA-induced p-PDK1 protein levels, it decreased phosphorylated Akt (pAkt)/Akt levels. Therefore, CLDN18 plays an important role in the pathogenesis of asthma and NAC diminishes airway inflammation by modulating CLDN18 expression [135].

CLDNs are major transmembrane protein components of TJs in the endothelia and epithelia. They enable TJs to maintain cell permeability, and also facilitate cell signaling via protein–protein interactions. CLDNs are implicated in airway inflammation following ozone exposure, suggesting that ozone affects TJ proteins via oxidative stress [136].

CLDN-4 reportedly functions as a paracellular sodium barrier, and is one of the three major CLDNs expressed in lung alveolar epithelial cells. Plasma CLDN-4 levels were significantly higher in patients with exacerbated bronchial asthma than in those without exacerbations. Plasma CLDN-4 levels were correlated with the eosinophil concentration, total immunoglobulin E, forced expiratory volume (FEV) 1% predicted, and the FEV1/forced vital capacity ratio. Moreover, lung tissue from OVA-induced mice exhibited significant increases in CLDN-4 transcripts and proteins, as well as more TJ breaks and more intense CLDN-4 staining. When CLDN-4 was downregulated by transfection with small interfering RNA, inflammatory cytokine expression, which was induced by the HDM allergen Der p1, was significantly increased. These findings raise the possibility that lung epithelial barrier proteins may be future treatment targets for asthma [137].

Inhaled corticosteroids are the most effective anti-inflammatory therapy currently available to treat persistent asthma. Corticosteroid therapy can also attenuate the increases in bronchial vascularity and edema frequently observed in patients with asthma. In our inflamed-airway mouse model, AHR and cytokine levels were reduced by corticosteroid treatment, and abnormal CLDN5 expression and endothelial integration were attenuated; this suggests that endothelial TJs may be therapeutic targets for decreasing airway inflammation. These findings indicate that the regulation of lung endothelial barrier function may be a promising novel therapeutic approach to treating asthma [138].

CLDN5 is critical for controlling endothelial cellular polarity and pericellular permeability. Mean plasma CLDN5 levels were higher in patients with COPD exacerbations than in those with stable COPD. The plasma CLDN5 levels measured in patients with COPD correlated with the duration of smoking. Plasma CLDN5 levels in patients with stable COPD were correlated with the predicted FEV1%d Plasma CLDN5 levels were not correlated with age. CLDN5 may be involved in COPD pathogenesis [139].

Respiratory syncytial virus (RSV) is the leading cause of lower respiratory tract infections in children worldwide. Although most of those affected develop a mild self-limiting illness, some develop severe acute lower respiratory infections and persistent airway disease. Exposure to ambient PM has been linked to asthma, bronchitis, and viral infections in many epidemiological studies. TiO_2_-NP exposure exacerbates RSV-induced adherens junction (AJ) complex dysfunction, and also exacerbates inflammation by generating ROS [140].

Airway epithelial barrier function is maintained by the formation of TJs and AJs. Inhalation of cigarette smoke (CS) causes airway epithelial barrier dysfunction and may exacerbate the pathogenesis of chronic lung diseases, such as asthma and COPD. Treatment with CS extract resulted in airway epithelial barrier dysfunction, and also downregulated many TJ and AJ proteins. LL-37 counteracted CS extract-induced reductions in transepithelial resistance and prevented the disruption of occludin and ZO-1. Therefore, using LL-37 to counteract airway epithelial barrier dysfunction may benefit patients with respiratory diseases such as asthma and COPD [141].

Wildfire smoke may induce acute pulmonary distress, particularly in high-risk groups such as the sick or elderly [142]. Wood smoke (WS) contains many of the toxic compounds found in CS, including polycyclic aromatic hydrocarbons, carbon monoxide, and free radicals. Exposure to CS is a well-established risk factor for respiratory diseases such as asthma and COPD. WS may facilitate the breakdown of alveolar structure via a p44/42 MAPK-dependent pathway, and chronic WS exposure may exacerbate respiratory diseases [142].

Wildfire smoke-extract inhibits autophagic flux and induces barrier dysfunction in the airway epithelium. As autophagy is a key regulator of cellular repair, viability, and inflammation, inhibiting autophagic flux may reduce the consequences of wildfire smoke exposure for individuals with pre-existing respiratory conditions [143].

CLDN5 is a critical component of the endothelial TJs, which control pericellular permeability. Acrolein, as one of the major irritants present in smoke, can induce acute lung injury, possibly by altering CLDN5 expression. Lung CLDN5 transcript and protein levels increased more in an acrolein-resistant cell line than in a susceptible cell line. In human endothelial cells, 30 nM acrolein increased CLDN5 transcript and p-FOXO1 protein levels. The phosphatidylinositol 3-kinase inhibitor LY294002 attenuated acrolein-induced increases in CLDN5 transcript levels. In addition, 300 nM acrolein decreased CLDN5 transcript levels and increased FOXO1 and CTNNB1 transcript levels. The levels of phosphorylation observed in these transcription factors were consistent with the changes observed in CLDN5 levels. Therefore, maintaining endothelial CLDN5 levels may be a novel clinical approach to treating acute lung injury [144].

Acrolein, an α/β-unsaturated aldehyde, is volatile at room temperature. It is a respiratory irritant present in environmental tobacco smoke and may be generated during cooking or at endogenous injury sites. Acrolein induces reactive airway dysfunction syndrome (RADS). Mouse model studies demonstrated that ROS, angiogenesis, and TJ proteins were involved in the development of RADS [145,146].

COPD pathogenesis is driven by the airway epithelium [147]. One important factor is a disease-related reduction in barrier function, which is potentiated by the dysregulation of TJ protein complexes [147]. The epithelial airway lining forms the initial barrier to environmental particles, such as inhaled CS, which is a major risk factor in COPD development [147]. The barrier is formed by epithelial junctions, which are interconnected structures that restrict the access of inhaled pathogens and environmental stressors [147]. Destruction of this epithelial barrier not only exposes subepithelial layers to hazardous agents in inspired air, but also alters the normal function of epithelial cells, which may, in turn, facilitate COPD development [147]. Disruption of epithelial junctions may lead to modulation of the signaling pathways involved in differentiation, repair, and proinflammatory responses. Epithelial barrier dysfunction may be particularly important in facilitating COPD development, because repeated injuries inflicted by CS, pathogens, and inflammatory mediators, together with impaired epithelial regeneration, may compromise barrier function [147].

DEPs elevate ROS, which can activate the nucleotide-binding oligomerization domain-like receptor containing pyrin domain 3 (NLRP3)-inflammasome. CS extract and DEPs increased the secretion of IL-1β in lung tissue, from both normal and elastase-induced emphysema samples. The level of secretion of IL-1β induced by CS extract and DEPs was higher in the elastin-induced emphysema than normal samples. NLRP3-inflammasome expression was increased by CS extract and DEPs in both the normal and elastin-induced emphysema samples, and was suppressed by NAC. In addition, the NLRP3-inflammasome was activated by DEPs in ex vivo tissue explants from an elastase-induced emphysema animal model, and this activation was also suppressed by NAC [147].

Chronic inflammation, oxidative stress, and proteolysis are all involved in COPD/emphysema pathogenesis. ApoA1 levels were significantly decreased in the lungs of patients with COPD, and in those of mice exposed to CS. ApoA1-overexpressing transgenic mice did not develop emphysema when they were exposed to chronic CS. Compared to control transgenic mice, those overexpressing ApoA1 exhibited attenuated lung inflammation, oxidative stress, metalloprotease activation, and apoptosis when exposed to CS. ApoA1 prevented CS-extract-induced translocation of Fas and downstream death-inducing signaling complex into lipid rafts, thereby inhibiting Fas-mediated apoptosis. Taken together, the data showed that ApoA1 overexpression attenuated CS-induced lung inflammation and emphysema in mice. Therefore, targeted augmentation of ApoA1 in the lung may have therapeutic potential by preventing smoking-related COPD/emphysema [148].

Acute exacerbations of COPD may occur due to air pollution, and ozone is an important pollutant. Vimentin, lactate dehydrogenase A, and triose phosphate isomerase levels were all decreased by both smoking and ozone exposure treatment. In contrast, TBC1 domain family 5 (TBC1D5) and lamin A levels were increased by both smoking and ozone exposure. treatment. Therefore, TBC1D5 may be a useful biomarker for ozone-induced lung injury in patients with emphysema [149].

## 2. Conclusions

Air pollution such as ozone, NO_2_, SO_2_, and PM is a major public health problem that exacerbates airway diseases such as asthma, COPD, and AR. Airway epithelial cells form the initial barrier to pollutants and play pivotal roles in the development of asthma and COPD. These cells are also potential therapeutic targets for maintaining airway integrity. Further basic and clinical research is warranted to identify environmental pollutants and novel therapeutic targets, as well as to elucidate their underlying mechanisms.

## Data Availability

Not applicable.
